# A rare case of concomitant sicca keratopathy and ipsilateral central facial palsy in Wallenberg’s dorsolateral medullary syndrome

**DOI:** 10.3205/oc000059

**Published:** 2017-03-07

**Authors:** Deborah De Bruyn, Elisabeth Van Aken, Kristien Herman

**Affiliations:** 1 Dpt. Ophthalmology, Ghent University Hospital & Ghent University, Ghent, Belgium; 2 Dpt. Ophthalmology, Sint-Elisabeth Hospital, Zottegem, Belgium

**Keywords:** central facial palsy, supranuclear facial palsy, sicca keratopathy, superior salivatory nucleus, medulla oblongata, dorsolateral medullary infarction, Wallenberg’s syndrome, subclavian steal syndrome

## Abstract

**Objective:** To describe a patient with a right-sided supranuclear facial palsy and concomitant sicca keratopathy of the right eye following right-sided dorsolateral medullary infarction.

**Methods:** Our patient underwent a complete ophthalmologic and neurologic examination including biomicroscopy, fundus examination, cranial nerve examination, Shirmer I test, and magnetic resonance imaging of the brain.

**Results:** A 61-year-old woman presented in emergency with a central facial nerve palsy on the right side and truncal ataxia. Neurologic assessment revealed a concurrent dysphagia, dysarthria, hypoesthesia of the right face, and weakness of the right upper limb. Magnetic resonance imaging of the brain showed an old left-sided cerebellar infarction, but a recent ischemic infarction at the level of the right dorsolateral medulla oblongata was the cause of our patient’s current problems. One month after diagnosis of the right-sided dorsolateral medullary syndrome, there were complaints of ocular irritation and a diminished visual acuity in the right eye. Biomicroscopy showed a sicca keratopathy with nearly complete absence of tear secretion on the Shirmer I test, but with normal eye closure and preserved corneal reflexes and sensitivity.

**Conclusion:** A dorsolateral medullary syndrome can have a variable expression in symptomatology. Our case is special because of the combination of an ipsilateral supranuclear facial palsy with normal upper facial muscle function together with an ipsilateral sicca keratopathy as a result of a nearly absent tear secretion. We hypothesized that the mechanism underlying the patient’s sicca keratopathy ipsilateral to the supranuclear facial palsy involved the superior salivatory nucleus, which is situated in the caudal pons inferiorly of the motor facial nucleus and is most probably affected by a superior extension of the infarcted area in the right medulla oblongata.

## Introduction

The dorsolateral medullary syndrome or also called Wallenberg’s syndrome is caused by an atherosclerotic or embolic occlusion of the vertebral artery or its branch, the posterior inferior cerebellar artery (PICA) [1]. A more uncommon cause is vertebral artery dissection [1]. The resulting infarction of the dorsolateral medulla oblongata induces damage to the trigeminal spinal nucleus and tract, spinothalamic tract, descending sympathetic fibers, inferior cerebellar peduncle, vestibular nuclei, and nucleus ambiguus [1]. The classic symptomatology consists of pain and temperature sensory deficits of the ipsilateral face and contralateral extremities, ipsilateral Horner syndrome and ipsilateral cerebellar signs [1], [2]. The variable clinical expression in Wallenberg’s syndrome is influenced by the extent and severity of the brainstem nuclei and spinal tracts involved and is thus dependent on the residual perfusion or collateral blood flow in areas at risk [1], [2]. 

The facial nerve or the seventh cranial nerve contains a motor portion and a sensory portion. The motor portion is responsible for the movement of the facial muscles (including the orbicularis oculi) in addition to the stapedius muscle in the middle ear, the stylohyoid, and the posterior belly of the digastric [3]. The motor nucleus of the facial nerve is situated in the caudal pons in proximity of the nucleus of the abducens nerve [4]. The upper facial motor nucleus supplying the upper half of the face receives input from both the ipsilateral and contralateral motor cortices whereas the lower facial nucleus has only contralateral input [3]. For this reason a supranuclear (or ‘central’) facial nerve lesion will cause a contralateral paralysis of the lower face with sparing of the upper facial musculature [3]. Infranuclear lesions as in a Bell’s palsy will usually result in a complete facial paralysis [3]. The parasympathetic portion of the facial nerve originates from the superior salivatory nucleus situated just inferior to the facial motor nucleus [4], [5]. The parasympathetic fibres supply the sublingual, submandibular, and lacrimal glands and are part of the nervus intermedius [5]. The second component of the nervus intermedius contains the sensory fibers subserving taste to the anterior two thirds of the tongue and somatic sensation to the external auditory meatus and postauricular region [5]. 

Here, we present a rare case with an interesting combination of ocular and neurologic symptoms including sicca keratopathy and ipsilateral central facial palsy as a part of ipsilateral Wallenberg’s syndrome. 

## Case description

**Figure 1:**
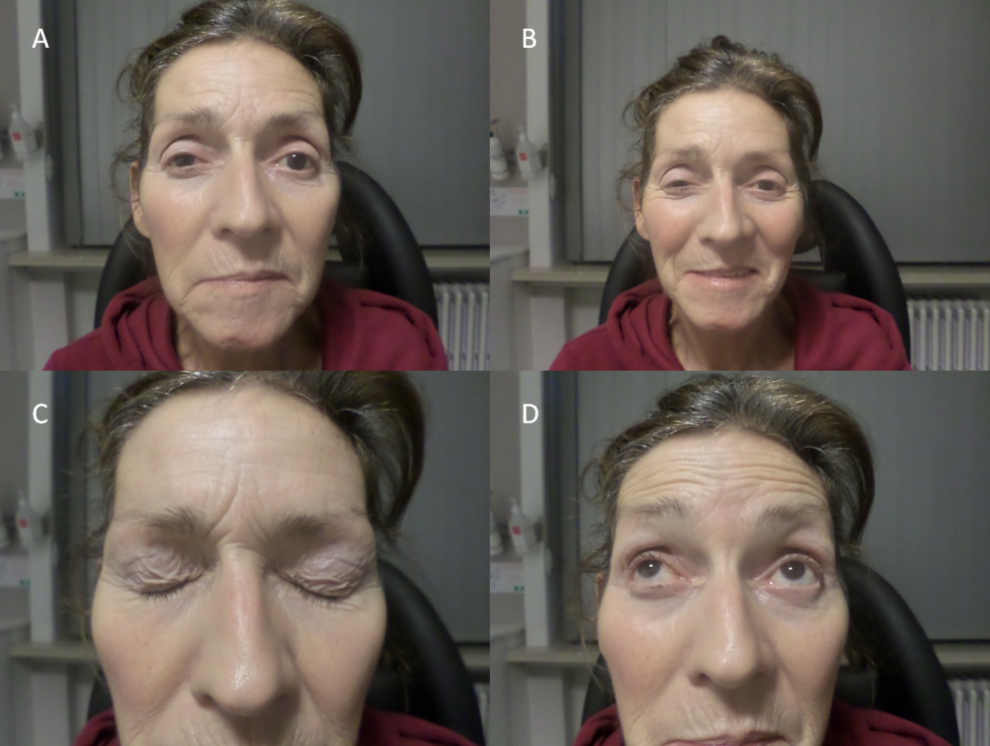
Right-sided central facial palsy of the lower part of the face. Note the drooping of the right mouth (A and B) with preserved eyelid closure (C) and upper facial muscle function when pulling up the eyebrows (D). Also note the right-sided Horner syndrome with mild ptosis and miosis (A).

**Figure 2:**
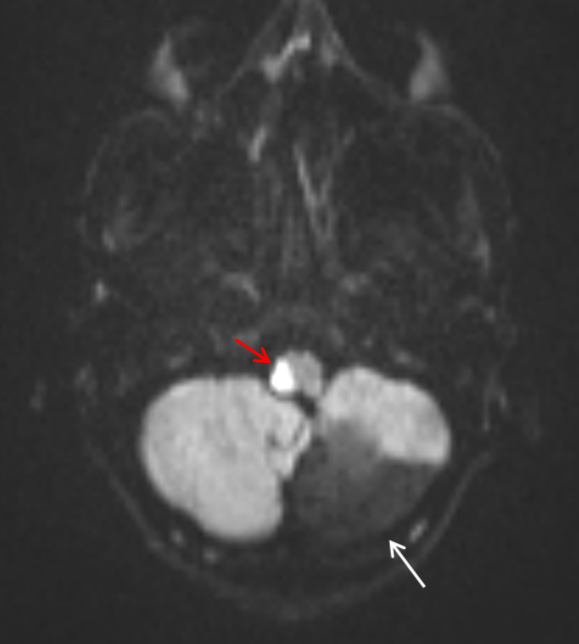
MRI imaging of the brain reveals diffusion restriction in the right medulla oblongata (red arrow) and an old infarction of the left cerebellum (white arrow).

A 61-year-old woman presented to the emergency department of our hospital with symptoms of occipital headache, vertigo with a tendency to fall to the right, nausea, drooping of the right mouth, and thermohypoesthesia of the right face and left extremities. In her medical history, we noted arterial hypertension, hypercholesterolemia, and peripheral arterial insufficiency. She also underwent an incomplete closure of a type 2 atrial septum defect in 2001. On presentation, her blood pressure was 180/120 mmHg with a regular pulse at 70 beats per minute. Neurologic examination revealed a right-sided central facial nerve palsy (Figure 1 (Fig. 1)), hypoesthesia of the right face, a mild right-sided partial Horner syndrome, and reduced strength in the right arm (Table 1 (Tab. 1)). Right-sided knee-heel testing was positive for dysmetria. A computed tomography (CT) scan of the brain without contrast revealed an old posterior cerebellar infarction on the left side. Subsequent computed tomography angiography (CTA) scan of the brain showed diffuse atheromatosis and a total occlusion of the left subclavian artery but without stenosis or obstruction in the carotid artery or vertebral artery system. Magnetic resonance imaging (MRI) of the brain two days after the onset of symptoms showed a diffusion restriction in the right dorsolateral medulla oblongata compatible with recent ischemia (Figure 2 (Fig. 2)). 

Cardiological work-up with echocardiography and duplex of the carotid arteries showed a persistent atrial septum defect with right-left shunt, a paroxysmal atrial fibrillation, and a subclavian steal syndrome as a result of a complete occlusion of the left subclavian artery. Fiberoptic laryngoscopy on otolaryngologic examination showed right vocal cord paralysis as a cause of her dysarthria. Due to the persisting dysphagia, a percutaneous endoscopic gastrostomy tube was placed for parenteral nutrition. 

**Figure 3:**
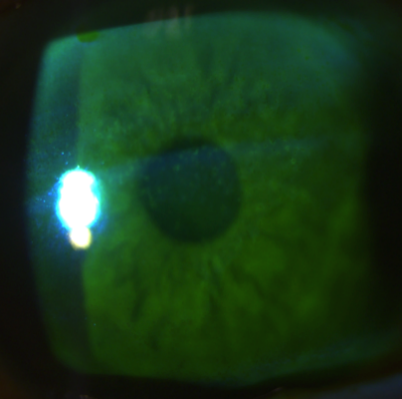
Sicca keratopathy of the right eye seen as superficial punctate fluorescein staining of the corneal epithelium

**Figure 4:**
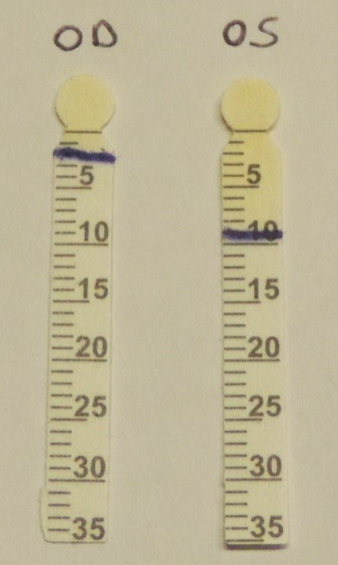
Shirmer I test reveals a nearly complete lack of tear secretion on the right side (1–2 mm) as compared to a normal tear secretion in the left eye (9–10 mm).

One month after the onset of neurologic symptoms, she complained of a foreign body sensation and a diminished visual acuity in the right eye. Best-corrected visual acuity on the right was 0.3 and 1.0 on the left. Biomicroscopy showed a pronounced sicca keratopathy of the right eye (Figure 3 (Fig. 3)). Corneal sensitivity was normal and symmetrical. The blink reflex was preserved. Ocular motility examination showed full-range eye movements without nystagmus. There was no lagophthalmos on eyelid closure. A Shirmer I test without anesthetic revealed a nearly absent tear production on the right side (1–2 mm) in comparison with a normal tear production on the left side (9–10 mm) (Figure 4 (Fig. 4)). The vertical lid opening was 9 mm on the right, 10 mm on the left. The levator function was normal and measured 12 mm on the right and 15 mm on the left. We started a local treatment with intensive lubrification with artificial tears and gel during the day and a thick ointment during the night to prevent corneal ulceration. 

## Discussion

Important risk factors for developing a dorsolateral medullary syndrome are vertebral artery dissection, which is more common in younger persons, and large artery atherosclerosis, which is more likely in older patients [1]. Another potential etiology that should be considered particularly in patients with arrhythmias, cardiac dysfunction, or valvular disease is embolic stroke originating from the heart [1]. Our patient exhibited multiple potential causes for developing an infarction of the medulla oblongata including diffuse atherosclerosis, an atrial septum defect with right-left shunt, an atrial fibrillation, and a subclavian steal syndrome. The atrial septum defect with right-left shunt can give rise to paradoxical emboli when a clot migrates from the right to the left side of the heart during a Valsalva maneuver. Subclavian-vertebral artery steal syndrome (SSS) is characterized by blood flow reversal in the vertebral artery due to significant stenosis or occlusion of the proximal ipsilateral subclavian artery or the innominate artery [6]. It is often asymptomatic and an incidental finding, but may also result in significant vertebrobasilar ischemia presenting mostly with transient ischemic attacks [6]. The role of the total occlusion of the left subclavian artery with subsequent subclavian steal syndrome in the development of the right medulla oblongata infarction is part of discussion in our patient as CTA scan of the brain could only detect diffuse atherosclerosis without any obstruction or stenosis in the carotid or vertebral artery system. 

As already mentioned above, Wallenberg’s dorsolateral medullary syndrome classically consists of pain and temperature sensory deficits of the ipsilateral face and contralateral extremities, ipsilateral Horner syndrome, and ipsilateral cerebellar signs [1], [2]. Clinical examination in our patient additionally showed an ipsilateral right facial nerve palsy, which has been described in rostral infarctions of the medulla oblongata with involvement of the caudal pons [1], [7]. The peculiar finding in our patient was the presence of an ipsilateral supranuclear or central facial palsy with drooping of the right mouth and preserved upper facial muscle function (including eyelid closure). Anatomically, we would rather expect an ipsilateral nuclear or infranuclear facial palsy as part of a dorsolateral medullary infarction. Possibly, the ipsilateral supranuclear facial palsy might be the result of involvement of the corticobulbar/corticofacial fibers after they crossed the midline as hypothesized by Maeshima et al. [8]. As our patient had a very high cardiovascular risk, another explanation for the supranuclear facial palsy would be the presence of a small second lesion not detectable on MRI imaging. 

However, patients with a central facial palsy do not present with sicca keratopathy. It is instead a common sign in patients with peripheral facial palsies where there is an inability to close the eyelids on the affected side or in patients with trigeminal nerve palsies and subsequent absent corneal sensitivity. We found two other cases reporting a neurotrophic corneal ulceration as a complication of Wallenberg’s syndrome [9], [10]. However, in these cases the authors hypothesized that the loss of corneal sensitivity was due to damage of the spinal trigeminal tract or nuclei [9]. Our patient exhibited normal corneal reflexes and sensitivity and had no lagophthalmos on eyelid closure. Yet, she had nearly absent tear secretion on the right side when performing a Shirmer I test. Notwithstanding the presence of a sympathetic innervation of the lacrimal gland, it is the parasympathetic innervation that controls lacrimal gland stimulation and is responsible for reflex tearing [11]. Despite the interruption of the descending sympathetic innervation in our patient causing a Horner syndrome on the right side, it is therefore less likely to conclude that this is the reason for the asymmetric Shirmer test. Taking all these arguments into consideration, this probably indicates that the infarction extended into the ipsilateral superior salivatory nucleus, which is located inferiorly of the facial motor nucleus and is responsible for the innervation of the sublingual, submandibular and lacrimal glands [4], [5]. There is no other single lesion that could be responsible for the combined symptomatology of a central facial palsy with reduced tear secretion. Even a more distal lesion, for example a lesion causing a partial infranuclear facial palsy (with sparing of the upper facial musculature) could anatomically never lead to damage to the parasympathetic fibers innervating the lacrimal gland because these fibers separate more proximally from the facial motor branches [5]. At presentation, our patient did not complain of a dry mouth. This is most probable due to the preserved innervation of the contralateral submandibular and sublingual glands. 

Rostral lesions of the medulla oblongata will also involve the nucleus ambiguus (the motor nucleus of the glossopharyngeal and vagus nerves) and present with severe dysphagia and hoarseness [1]. Our patient showed right vocal cord paralysis and a severe dysphagia with the necessity to place a gastrostomy tube. In addition, our patient also displayed vertigo and cerebellar ataxia which can be a result of involvement of the vestibular nuclei and cerebellar outflow tracts in the caudal medulla [1]. In our patient, imaging of the brain showed an old infarction of the left cerebellum but this cannot explain the acute onset symptoms of vertigo and ataxia. 

## Conclusion

We report a clinical case demonstrating the atypical combination of a right-sided Wallenberg’s dorsolateral medullary syndrome together with an ipsilateral central facial palsy and additional sicca keratopathy. We hypothesize that the mechanism underlying the patient’s sicca keratopathy ipsilateral to the supranuclear facial palsy involves an extension of the medullary infarction into the superior salivatory nucleus located in the caudal pons. Finally, it is important that clinicians are aware that even a central facial nerve palsy can bear a sicca keratopathy on the affected side if the parasympathetic nucleus of the facial nerve is affected. All patients with a central facial nerve palsy and complaints of a red and irritated eye need ophthalmologic examination to exclude sicca keratopathy. Proper treatment consisting of extensive lubrification is needed to prevent corneal ulceration and scarring. 

## Notes

### Competing interests

The authors declare that they have no competing interests.

### Informed consent

Informed consent was obtained from the patient for the explicit use of the images and medical information. 

## Figures and Tables

**Table 1:**
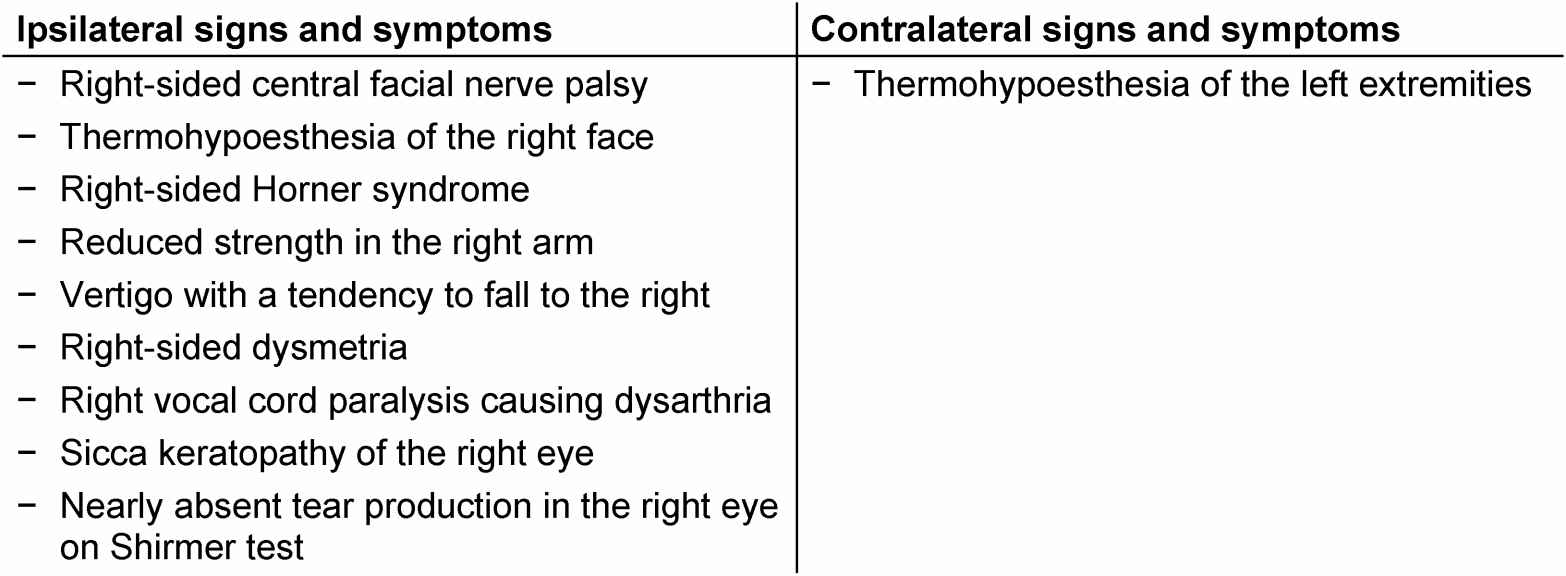
A list of all the signs and symptoms presented by our patient suffering a right-sided dorsolateral medullary infarction
